# A Rare Case of Emphysematous Osteomyelitis With Concurrent Septic Arthritis

**DOI:** 10.7759/cureus.64898

**Published:** 2024-07-19

**Authors:** Omar Brijawi, Evan Hartman, Sumit Sharma, Mark Herbert, Ruth Mullowney-Agra

**Affiliations:** 1 Internal Medicine, Mount Carmel Health System, Grove City, USA; 2 Internal Medicine, Wright State University Boonshoft School of Medicine, Dayton, USA; 3 Infectious Disease, Mount Carmel Health System, Grove City, USA

**Keywords:** pumice stone sign, e. coli, septic arthritis, intermedullary gas, emphysematous osteomyelitis

## Abstract

A rare and possibly fatal infection of the bone called emphysematous osteomyelitis (EO) is caused by the presence of intraosseous gas due to gas-forming organisms. Common gas-producing organisms are in the *Enterobacteriaceae *family or are anaerobes. This gas within bones is most frequently detected using computed tomography (CT) imaging, and prompt diagnosis is important due to the high mortality rate. We present a 76-year-old male who complained of altered mental status, right upper and lower extremity weakness, and lower back pain. The MRI of the lumbar spine showed moderate edema in L3 and L4, with fluid in L3-L4 and L4-L5 concerning discitis/osteomyelitis. A CT-guided biopsy of L3/L4 was then performed by interventional radiology, revealing air present in the L3 and L4 vertebral bodies. Bone cultures from the L3 and L4 vertebra were later positive for *E. coli *that was susceptible to all tested antibiotics, and this was consistent with a diagnosis of vertebral EO. The infectious disease team recommended a six-week course of intravenous ceftriaxone. During the patient’s hospital stay, he also developed a septic right knee joint positive for *E. coli, *alongwith the concurrent vertebral EO.

## Introduction

Emphysematous osteomyelitis (EO) was first described in 1981, when computed tomography (CT) imaging was used to describe intraosseous gas in the medullary cavity of the affected bone [1]. With only 45 cases reported throughout the literature, EO is an exceedingly rare diagnosis [2]. Underlying comorbidities such as diabetes mellitus and malignancy are common predisposing factors that contribute to EO, while common causative organisms include Enterobacteriaceae family members and anaerobes [3,4]. Prompt diagnosis and initiation of treatment are imperative due to EO’s life-threatening potential. One analysis described the mortality among cases as upwards of 32% [3]. In this case report, we discuss a 76-year-old male diagnosed with emphysematous osteomyelitis of the spine caused by *E. coli*.

## Case presentation

A 76-year-old male presented to the emergency department with flu-like symptoms, loss of appetite, nausea, vomiting, cough, knee pain, and back pain for the past four days. The patient’s medical history was notable for hypertension, gastroesophageal reflux disease (GERD), right tibial IM nail procedure, and osteoarthritis. On arrival at the emergency department, the patient was hemodynamically stable with significant laboratory findings of a WBC of 10.4, thrombocytopenia of 101k, and creatinine of 2.63. The chest X-ray was significant for a left basilar opacity, and he was subsequently placed on ceftriaxone and azithromycin. Blood cultures were not obtained before ceftriaxone and azithromycin were initiated. 

**Figure 1:**
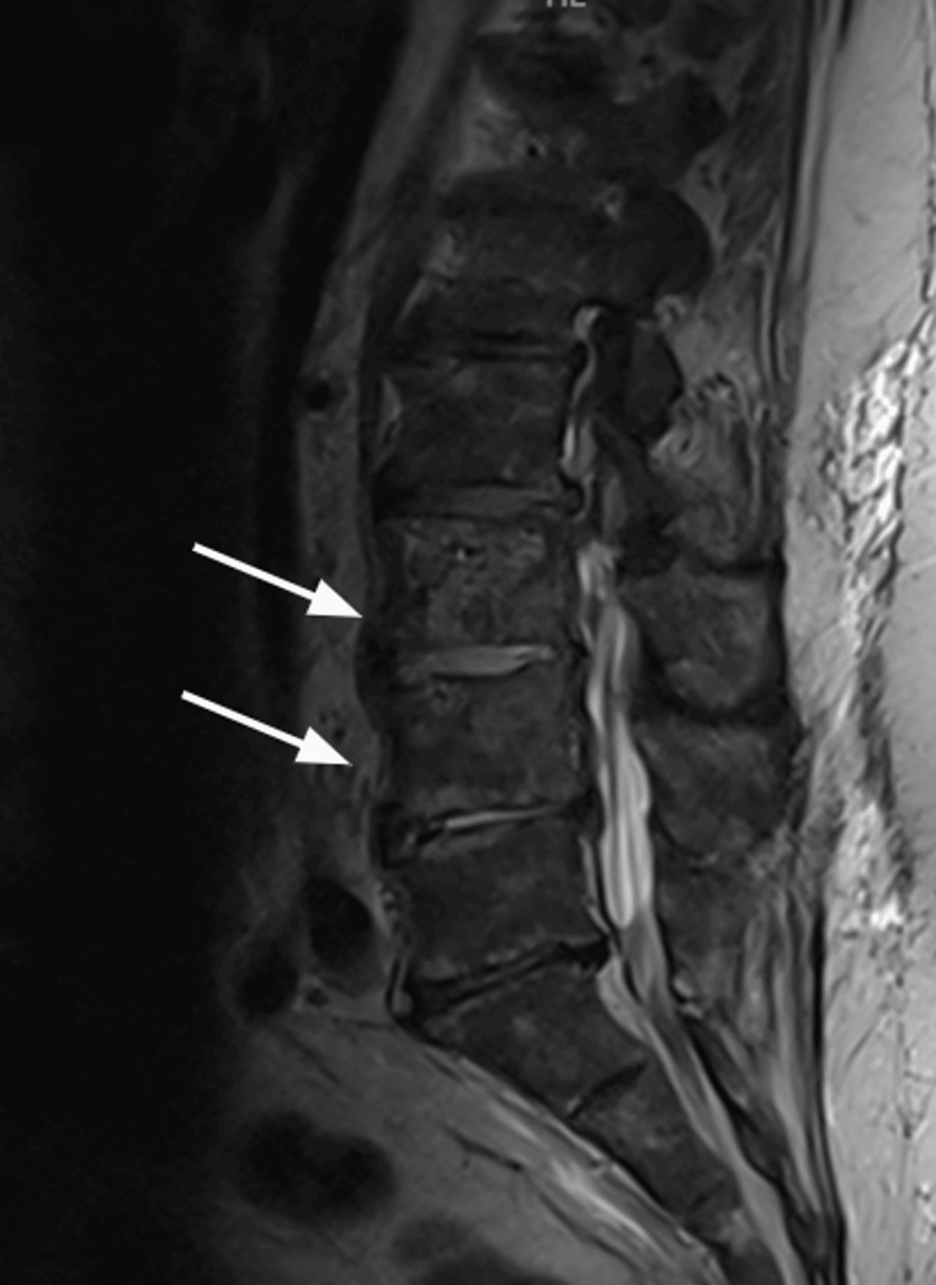
MRI SAG T2 of the lumbar spine showing moderate marrow edema in the L3 and L4 vertebrae with fluid in the L3-L4 and L4-L5 discs concerning for discitis/osteomyelitis.

By day 3 of hospitalization, the patient began developing altered mental status, right upper and lower extremity weakness, and an inability to lift his right lower extremity. An MRI of the brain ruled out any evidence of an acute infarction. MRIs of his cervical and thoracic spine were only significant for degenerative changes and mild central stenosis. The MRI of his lumbar spine was significant for moderate edema in L3 and L4, with fluid in L3-L4 and L4-L5 concerning discitis/osteomyelitis, but no epidural collection was identified (Figure 1). He was also noted to have a small psoas abscess on the left, measuring 13 mm × 9 mm.

**Figure 2:**
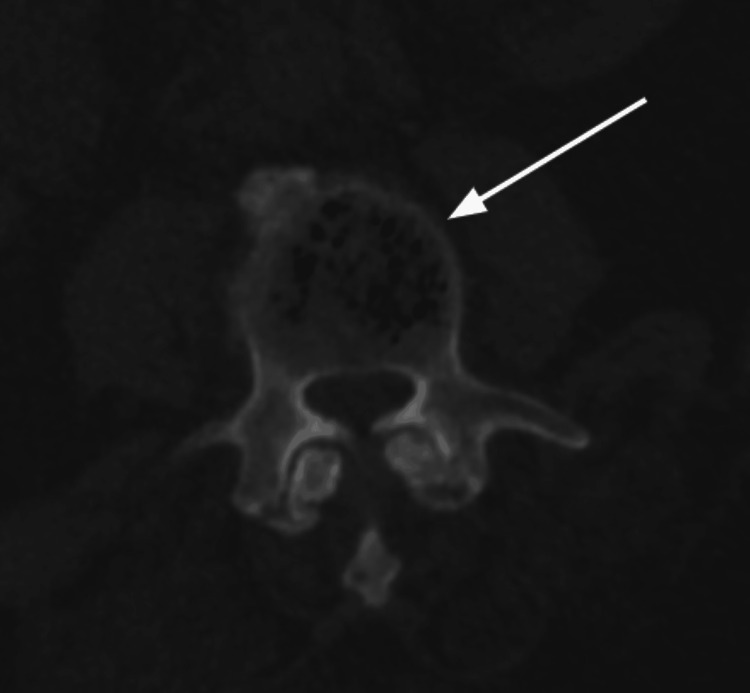
CT image-guided biopsy visualization of the “pumice stone sign” in the L3 vertebra corresponding with emphysematous osteomyelitis.

Neurosurgery advised against any surgical intervention at the time. In order to obtain optimal microbiological data from a vertebral body biopsy and culture, antibiotics were discontinued as the patient was not septic. During the biopsy, a CT scan was done, which showed air foci in the L3 and L4 vertebral bodies, confirming the diagnosis of emphysematous osteomyelitis (Figure 2).

The patient's bone culture returned pan-sensitive to *E. coli*, and he was placed on a total of six weeks of intravenous ceftriaxone. Subsequently, the patient developed a right knee effusion along with recurrent fevers, and a right knee joint aspiration was significant for septic arthritis with a white blood cell count of 31,000 and a growth of pan-sensitive *E. coli*. 

The patient was discharged to a skilled nursing facility with a plan to continue the full six weeks of ceftriaxone for his emphysematous osteomyelitis/discitis along with his septic arthritis of his right knee.

## Discussion

Intraosseous gas observed within the axial and extra-axial skeleton is pathognomonic for emphysematous osteomyelitis [5]. E. coli was the causative agent in this case, but it is important to consider the various causes of intraosseous gas to configure the best treatment. A review of EO cases mentions that there is no predominance of sex, and the median age is around 51.7 years [6]. EO does appear to occur more among patients with comorbid medical conditions such as diabetes and malignancy. Other predisposing factors include enteritis, alcohol abuse, and hematological disorders such as sickle cell disease [7].

Radiologic imaging plays a crucial role in the diagnosis of EO, with computed tomography imaging being the most commonly used imaging modality. CT and MRI were utilized to detect intraosseous gas within this patient's vertebrae, but X-rays and ultrasounds did not play a role in the diagnosis. In EO, the presence of intraosseous gas is described as the “pumice stone sign” - a cluster of three or more foci of intramedullary gas [8]. X-ray films are capable of detecting air pockets within bones, but ultrasound is not as helpful. Magnetic resonance imaging is also capable of detecting gas, but further research is needed to determine how it correlates to CT imaging for intraosseous gas detection [9]. EO has a broad differential diagnosis that includes subchondral cysts, ischemic necrosis, and penetrating wounds [10]. Additional items on the differential include non-infectious causes such as degenerative disc disease, neoplasm, and osteonecrosis [2]. It is important to note that gas distribution patterns should be used in addition to the differential diagnosis. The gas distribution pattern of gas-forming bacteria in EO is bubbly and irregular, while a linear/well-defined pattern that does not alter surrounding tissue and contains evenly spaced trabeculae is more suggestive of degenerative disease [11]. CT imaging utilization and pattern recognition are critical in diagnosing EO to ensure the prompt initiation of management.

Although EO occurred within the vertebra in this case report, EO is not solely confined to affecting the vertebra. One review observed that the vertebral column was the most common site of EO at 57.1%; the second most common site was the femur at 24.5%, followed closely by the pelvic bones at 24.4% [7]. An older review also listed the vertebral column, pelvis, and femur as common sites of EO [10]. One way of spreading the causative agent is contiguous via an extension of a soft tissue infection or surgery [12]. The other transmission mode to the bone is hematogenous through the blood supply. Intraosseous gas formation in EO has been attributed to bacteria within the Enterobacteriaceae family or anaerobes. Common causative bacteria include E. coli and Klebsiella pneumonia, while the most common causative anaerobe is Fusobacterium necrophorum [2,5,11-13]. Postsurgical infectious causative organisms vary slightly because Staphylococcus aureus, non-hemolytic streptococci, Pseudomonas, and enterococci are likely causes [14]. Isolating the causative organism will help to narrow the antibiotic coverage appropriately.

Due to the high mortality rate of EO, aggressive treatment with antibiotics is recommended to prevent other complications, such as the destruction of the bone. Empiric treatment of Enterobacteriaceae family bacteria and anaerobes should be initiated once EO is suspected. Antibiotic treatment is recommended for at least four to six weeks [2,5,14]. The treatment recommendation for EO is similar to that for osteomyelitis treatment because of the rarity and lack of a treatment conclusion for EO, but a longer duration may be necessary depending on the patient’s condition. Surgery is usually recommended for patients who fail to respond to antibiotic treatment or have complications such as necrosis or abscesses [5,8,14].

Due to its high mortality rate, EO is considered an aggressive condition. One study of 25 cases calculated the mortality rate of EO to be about 32% [3]. Another review, which analyzed 41 cases, found the calculated mortality rate to be 34% [7]. Additionally, two studies observed that the mortality rate of vertebral EO approaches up to 50% [2,3]. Delays in diagnosing EO could play a factor in the high mortality.

## Conclusions

We report a case of a 76-year-old male diagnosed with vertebral emphysematous osteomyelitis caused by *E. coli*. The patient was treated with six weeks of intravenous antibiotic therapy using ceftriaxone. Intraosseous gas detected on CT imaging is highly suggestive of EO. Considering the high mortality of EO, aggressive antibiotic therapy and potential surgery for complications are the current treatment practices. Additional research is needed to establish more conclusive treatment recommendations.
